# Sexual Dimorphism in the Expression of Cardiac and Hippocampal Renin-Angiotensin and Kallikrein–Kinin Systems in Offspring from Mice Exposed to Alcohol during Gestation

**DOI:** 10.3390/antiox12030541

**Published:** 2023-02-21

**Authors:** Gabriel Almeida da Silva, Allan Luís Barboza Atum, Leonardo Paroche de Matos, Guilherme Rabelo Nasuk, Bruna Calixto de Jesus, Telma Luciana Furtado Gouveia, Ovidiu Constantin Baltatu, Stella Regina Zamuner, José Antônio Silva Júnior

**Affiliations:** 1 Medicine Department, Universidade Nove de Julho (UNINOVE), Rua Vergueiro 249, Liberdade, São Paulo 01504-001, SP, Brazil; 2 Odontology Department, Ágora Faculty, Av. Sorrento, Campo Novo do Parecis 78360-000, MT, Brazil; 3 Biomedicine Department, Finaci Faculty, Rua São Joaquim, 352 Liberdade, São Paulo 01508-901, SP, Brazil; 4 Center of Innovation, Technology and Education (CITE), Anhembi Morumbi University, Anima Institute, Sao Jose dos Campos Technology Park, Sao Jose dos Campos 12247-016, SP, Brazil

**Keywords:** prenatal alcohol exposure, renin–angiotensin system, kallikrein–kinin system, hippocampus, heart, oxidative stress

## Abstract

Prenatal alcohol exposure (PAE) impairs fetal development. Alcohol consumption was shown to modulate the renin–angiotensin system (RAS). This study aimed to analyze the effects of PAE on the expression of the renin–angiotensin system (RAS) and kallikrein–kinin system (KKS) peptide systems in the hippocampus and heart of mice of both sexes. C57Bl/6 mice were exposed to alcohol during pregnancy at a concentration of 10% (*v*/*v*). On postnatal day 45 (PN45), mouse hippocampi and left ventricles (LV) were collected and processed for messenger RNA (mRNA) expression of components of the RAS and KKS. In PAE animals, more pronounced expression of *AT1* and *ACE mRNAs* in males and a restored *AT2 mRNA* expression in females were observed in both tissues. In LV, increased *AT2*, *ACE2*, and *B2 mRNA* expressions were also observed in PAE females. Furthermore, high levels of H_2_O_2_ were observed in males from the PAE group in both tissues. Taken together, our results suggest that modulation of the expression of these peptidergic systems in PAE females may make them less susceptible to the effects of alcohol.

## 1. Introduction

Alcohol use disorders in adults are well evidenced in the literature, especially related to the central nervous system (CNS) and heart. In addition, observational evidence pointed out the benefits of low alcohol intake concerning mental and ischemic diseases [1,2,3]. Nonetheless, there is a consensus that drinkers bearing cardiovascular and brain diseases show improved outcomes with reduced alcohol consumption [4,5]. Thus, the recommendations regarding moderate consumption should be individualized to reflect the risks of alcohol and its effects on various chronic diseases [6]. Overall, increased volumes and patterns of alcohol consumption correlate positively with disease risks [7,8].

An estimated 32.1% of women of childbearing age consumed alcohol [9]. Moreover, it is estimated that the prevalence of alcohol use during pregnancy in the world population is 9.8% [10]. When consumed by pregnant women, alcohol is particularly harmful to the developing fetus [11,12]. Prenatal exposure to alcohol (PAE) is a challenging public health problem, and alcohol can induce, in the fetus, severe physical and mental impairments described as fetal alcohol syndrome (FAS) [13] or fetal alcohol spectrum disorders (FASD) [14]. Early studies indicated that alcohol use during gestation is nearly equally distributed between maternal and fetal tissues, and the hippocampus is mainly affected by PAE [15]. Authors have shown that hippocampal cell number is altered after PAE [16], inducing significant loss of hippocampal cells in the third trimester of human pregnancy [17]. In rodents, the equivalent of the third trimester period occurs within the first ten postnatal days, and PAE can have deleterious effects on hippocampus structure and function [18]. Furthermore, in adult mammals, PAE affects hippocampal neurogenesis [19]. PAE also severely affects the heart, leading to congenital cardiac diseases [20] These dysfunctions mostly result in miscarriage or are detected at birth in survival children [12]. However, the effects of ethanol on the heart of adults with PAE are little explored. A study [21] reported that PAE could alter the myocardial contractile function and contribute to the development of postnatal cardiac dysfunction. The authors revealed that alcohol exposure increased intracellular Ca (2+) load and apoptosis in adulthood. We recently reported that PAE could modulate the mRNA expression of components of nine cardiac transduction signal pathways related to heart diseases in male mice [22].

The renin–angiotensin system (RAS) plays a central role in the development and progression of cardiovascular disorders [23]. However, the brain expression of RAS is related to blood pressure control and elicits new functions of Ang II and other RAS components in cell signaling [24]. First, the enzymatic action of renin (REN) over the precursor angiotensinogen generates angiotensin I (Ang I), a decapeptide with low biological action. Then, Ang I is converted to Ang II by the angiotensin-converting enzyme (ACE) that removes the His-Leu dipeptide from the C-terminal portion of the angiotensin molecule. Ang II presented high affinity for the angiotensin receptors AT1 and AT2, although there are differences in the outcome of their bindings. The AT1 receptor mediates Ang II-induced vasoconstriction, proliferation, oxidative stress, inflammation, and extracellular matrix remodeling. The activation of the AT2 receptor produces opposite effects, providing a protective action [20]. Angiotensin-converting enzyme 2 (ACE2), a protective component of the RAS, catalyzes the conversion of Ang II into Ang 1-7 to counterbalance ACE activity [25].

Angiotensin II (Ang II) has been the main point of interest in investigations into the role of the RAS in the CNS, as it is a peptide related to synaptic plasticity, and it blocks long-term potentiation in the hippocampus. Another point of interest is the ACE, a therapeutic target to control the effects of the RAS, which is responsible for degrading bradykinin, the major effector of the kallikrein–kinin system (KKS) [26]. This peptidergic system is the counterpart of the RAS in blood pressure control. Bradykinin is formed by the action of tissue kallikrein (KLK) over kininogen and binds in two transmembrane receptors named kinins B1 and B2 receptors [27]. While B1 has low expression under physiological conditions, augmented by inflammatory signals, the B2 receptor is ubiquitously expressed. Moreover, both receptors are expressed in many tissues, including the hippocampus, where it participates in neuroinflammation processes [27].

One of the major players in neuroinflammation is the RAS, which, when activated by inflammation, increases sympathetic drive, potentially exacerbating heart conditions [28]. Neuroinflammation is a common feature of alcohol-induced brain damage and can cause neurodegeneration [29]. Previously, [30] showed that RAS activation is inversely related to alcohol intake, but accumulating evidence suggested that alcohol stimulates the RAS in animals and humans. Increased RAS activity is found in both light and heavy drinkers [31,32]. Recently, [33] reported that adolescents with PAE were at risk for low-grade systemic inflammation. However, whether the peptidergic RAS and KKS contribute or protect against PAE effects still needs clarification. So, since the hippocampus and heart tissues have been linked to offspring’s neuropsychological and cardiovascular disorders caused by PAE, we designed this study to investigate the possible effect of alcohol on the gene expression of two neurohormonal systems, the renin–angiotensin system (RAS) and the kallikrein–kinin system (KKS) peptide systems, in the hippocampus and heart of adult mice of both sexes. We purposefully avoided posing a directional hypothesis about the impact outcomes because of the scarcity of data on the PAE effects on the RAS or KKS in the tissues of interest. Thus, our study analyzed the mRNA expression of components of the vasoactive renin–angiotensin and kallikrein–kinin systems and ROS content in samples of the hippocampus and myocardium of mice of both sexes with PAE in early adulthood (postnatal day 45, PN45).

## 2. Materials and Methods

### 2.1. Animals

Fifteen isogenic mice (10 females and 5 males) of the C57Bl/6 strain (weighing 17–22 g) obtained at the Animal Facility of the Universidade Nove de Julho were used to generate the offspring. The animals were confined in appropriate plastic boxes under a light cycle (light/dark cycle, 12 h/12 h) with temperature (21 ± 2 °C) and humidity controlled. The access to food, water, or alcoholic solution was ad libitum as described by [22,34,35]. The females were randomized into two groups: the control group—CT (n = 3) and the prenatal alcohol exposure group—PAE (n = 7). Males were used only as breeders and removed from boxes after mating. All progenitors were euthanized after weaning. Offspring were randomly assigned to the control (n = 20) or PAE group (n = 20), and each group of mice was equally subdivided into two subgroups (n = 10 animals each) with male and female mice, avoiding siblings in the same experimental group.

### 2.2. PAE Protocol

The PAE protocol was performed previously by [22,34,35]. Briefly, the protocol started with a sensitization period (SP1 to SP15) of female mice, and it was established in 3 steps. In the first 4 days (SP1 to SP4), each female received a 0.1% aqueous saccharin solution with a new solution available every two days to avoid fungal proliferation. Then, a 2% alcoholic solution was introduced to the females on days SP5 and SP6 days. Then, on days SP7 and SP8, a 5% EtOH solution was offered, and after this period, the animals received, for seven days (SP9 to SP15), a 10% EtOH solution with 0.1% saccharin. The low amount of saccharin added to the water was used to mask the bitter taste of alcohol, making the solution more palatable.

The males were confined with the females for mating, and the animals had access to the 10% EtOH alcoholic solution with 0.1% saccharin. From the gestational period (gestational day 1—GD1- to GD19/21) until the 10th day after delivery (postnatal day 10—PN10), each female mother received the alcoholic solution at 10% EtOH/0.1% saccharin. As of PN11, the alcohol desensitization protocol began, so a 5% EtOH-sweetened solution was available to each female. On day PN13, there was a reduction to 2% EtOH solution, and from day PN15 to PN21, they received 0.1% saccharin solution. Then, the females received only filtered water from day PN22 until weaning (PN30) [22,34,35]. The control groups received the same proportion of water and saccharin at each period established to PAE but without alcohol sensibilization and administration.

### 2.3. Biological Samples

At PN45, male and female offspring were euthanized by decapitation under isoflurane anesthesia. After craniotomy, the brain was collected, and the hippocampus was dissected in an iced plate. Next, a left thoracotomy was performed, the heart was exteriorized, and the left ventricle (LV) was dissected and washed to remove blood content. The hippocampus and LV samples from animals of the control and PAE groups were then snap-frozen and stored at −80 °C. For the qPCR protocol, we used 5 animals per group, and 4 animals per group were used for ROS measurements. As the number of animals born per gestation was unpredictable, if there were surplus animals, they were also euthanized, and the organs were collected for further analyses unrelated to this study.

### 2.4. Quantitative PCR

#### 2.4.1. RNA Extraction

Hippocampal and LV samples from both groups (n = 5 per group) were homogenized with Trizol^®^ Reagent (Life Technologies, Carlsbad, CA, USA) to extract total RNA, according to the manufacturer’s instructions. The total RNA quantification of each sample was obtained using the NanoDrop ND-2000 spectrophotometer (NanoDrop Products, Wilmington, DE, USA). Only samples free of contaminants (A260/A230 ~1.8) and proteins (A260/A280 = 1.8–2.0) were used. One µg of total RNA was incubated with 1 unit of DNase I/RNase-free to eliminate genomic DNA contamination, and the samples were kept at −80 °C.

#### 2.4.2. Reverse Transcription

Reverse transcription was carried out using the SuperScript IV Reverse Transcriptase Kit (Invitrogen, Carlsbad, CA, USA). Initially, extracted RNA was added to a solution containing 1 µL of 50 µM Oligo d(T)20 Primer, 1 µL 10 mM dNTP mix, and 1 µL of DNase I/RNase Free (Invitrogen, Carlsbad, CA, USA). Then, the samples were incubated in a thermocycler for 5 min at 65 °C and added 7 µL of a solution containing 1 µL of Ribonuclease Inhibitor, 1 µL of SuperScript^®^ IV Reverse Transcriptase (200 U/µL), 1 µL 100 mM DTT, and 4 µL 5x SSIV Buffer (Invitrogen, Carlsbad, CA, USA). The resulting solution was incubated for 10 min at 55 °C and then for 10 min at 80 °C. Next, 1 µL of RNase H (Invitrogen, Carlsbad, CA, USA) was added and incubated for 20 min at 37 °C to remove residual RNA. After the reaction, the cDNA samples were kept at −20 °C until qPCR was performed.

#### 2.4.3. Quantitative Real-Time Polymerization Chain Reaction (PCR)—qPCR

Amplification and data acquisition were performed using the SYBR Green method in a Quantstudio™ 5 System equipment (Applied Biosystems, Carlsbad, CA, USA). The samples were mixed with PowerUp SYBR Green Master Mix 2x, and specific primers and nuclease-free water were added, totalizing a final volume of 20 µL. The reactions were incubated at 95 °C for 20 s and passed through 40 thermal cycles at 95 °C for 3 s and 60 °C for 30 s. All primers were designed using Primer-BLAST [36], purchased from Exxtend Biotecnologia Ltd.a. (Paulínia, São Paulo, Brazil). The primer sequences are shown in Table S1 (Supplementary Materials).

The reactions were submitted to the same conditions, and all experiments were repeated 3 times. The data, expressed in CT value, referred to the number of PCR cycles required for the fluorescent signal to reach the detection threshold. The differentially expressed genes were normalized by the expression of the housekeeping gene *18S subunit of the ribosomal RNA*, the expression of which was unaltered under the experimental conditions. The QuantStudio™ Design & Analysis software version 1.3.1 (Applied Biosystems, Carlsbad, CA, USA) was used for data processing. The ΔCT values were determined by subtracting the mean CT value of the target gene mRNA from the mean CT value of the *18S rRNA* housekeeping gene. The 2^−ΔΔCT^ parameter was used to represent the relative expression data.

### 2.5. ROS Measurement

The hydrogen peroxide content in hippocampal and myocardial homogenates was measured using a colorimetric assay according to the manufacturer’s instructions (Abcam, Cambridge, UK, ab102500). Briefly, the tissues (n = 4 per group) were homogenized using the assay buffer, and dilutions of the sample homogenates were incubated with a reaction mix containing the peroxidase fluorogenic substrate OxiRed probe in a 1:1 ratio. The absorbance was measured at 570 nm.

### 2.6. Statistical Analysis

The gene expression results were coded blindly per group, and the statistician remained blind to the coding allocation until the analysis was completed. Statistical calculations were performed using the IBM Corp. application. IBM SPSS Statistics for Windows, Version 25.0 (Armonk, NY, USA: IBM Corp., 2017). To verify normality and error variances, the Shapiro–Wilk test was used. First, Student’s t-test was performed for comparison between groups, followed by the Mann–Whitney test, with a significance of ≤0.05. Then, Student’s t-test was performed to compare gene expression and, when necessary, supplemented with the Welch correction test. A *p*-value ≤ 0.05 was considered significant, and results were expressed as mean ± standard error of the mean (SEM).

## 3. Results

Hippocampal and myocardial samples from control and PAE groups were collected from 45-day-old animals of both sexes (PN45). Then, the tissues were processed and submitted to mRNA quantification of eight genes coding for components of the RAS and the KKS (AT1 and AT2 receptors, renin, ACE, ACE2, kinins B1 and B2 receptors, and KLK genes). The myocardial expression data of RAS genes in the Control and PAE animals of both sexes are shown in Table 1.

Data referring to hippocampal gene expression of RAS components in animals from experimental groups of both sexes are shown in Table 2.

Our data suggested significant increases in *AT1* expression in the PAE males and females in the hippocampus (HC) and myocardium (MC) compared to the respective controls; however, *AT1* mRNA expression was sharply increased (2.9-fold in the HC and a 1.9-fold in the MC, *p* ≤ 0.001) in males with PAE compared to the control. PAE females presented increases of 1.2-fold and 1.7-fold in *AT1* mRNA expression in the MC and HC, respectively. Comparing *AT1* mRNA expression between animals with PAE, males presented a 2.1-fold increase in MC, and an even higher difference was observed in the HC (2.9-fold) compared to females. On the contrary, angiotensin *AT2* receptor gene expression was increased in the PAE females than in the control (2.1-fold in the HC and 1.6-fold in MC). An increased *AT2* expression was also seen in PAE males compared to the control (1.3-fold in both tissues), although expression levels were lower than in the PAE female group. A comparison between PAE groups revealed a 2-fold increase in *AT2* mRNA expression in PAE females than in males. Furthermore, the basal expressions of *AT1* mRNA in the hippocampus and myocardium were lower in control females than in males (MC: 3.48 ± 0.39 vs. 4.72 ± 0.51; HC: 1.92 ± 0.53 vs. 3.43 ± 0.41; *p* ≤ 0.05). In contrast, both tissues presented higher *AT2* mRNA expression in control females than in males (MC: 1.57 ± 0.31 vs. 0.92 ± 0.21; HC: 1.01 ± 0.18 vs. 1.34 ± 031; *p* ≤ 0.05).

Messenger RNA expression of the *renin* gene in the control groups in both tissues was unaltered (MC: 3.43 ± 0.28 in males and 2.96 ± 0.21 in females; HC: 2.71 ± 0.72 in males and 2.19 ± 0.83 in females). However, PAE females had lower *REN* mRNA expression than PAE males in the myocardium (3.53 ± 0.30 vs. 5.82 ± 0.86), with no changes observed in the hippocampus (2.94 ± 0.68 in PAE males and 2.63 ± 033 in PAE females). PAE increased the male and female *ACE* mRNA expressions; however, this augmentation was higher in PAE males than in females in the hippocampus (1.3-fold) and myocardium (1.8-fold). Regarding the *ACE2* mRNA expression, PAE induced an mRNA increment, regardless of sex, compared to their respective controls. However, in both tissues, PAE females presented higher *ACE2* expression than PAE males (HC: 4.87 ± 0.23 vs. 2.93 ± 0.57; MC: 4.19 ± 0.46 vs. 3.28 ± 0.34, *p* ≤ 0.05). Females from the control group presented increased *ECA2* mRNA levels compared to control males (1.1-fold in the HC and 1.9-fold in the MC). Control *ECA* mRNA presented similar expressions in both tissues (MC: 3.79 ± 0.81 in males and 3.99 ± 0.54 in females; HC: 4.12 ± 0.84 in males and 3.64 ± 0.27 in females).

**Figure 1:**
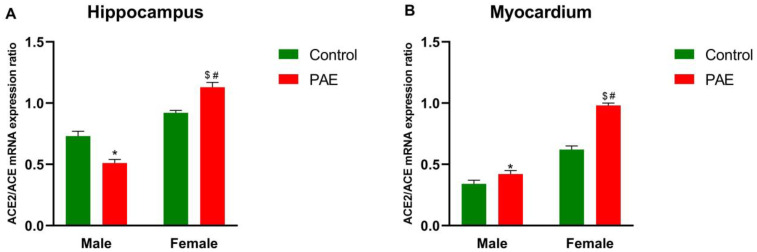
Effects of PAE on the ACE2/ACE mRNA ratio in the hippocampus (**A**) and the myocardium (**B**) in animals of both sexes with PAE. Data are shown as mean ± SEM of 5 animals per group. * *p* ≤ 0.001 vs. Control male; ^$^  *p* ≤ 0.05 vs. Control female; ^#^  *p* ≤ 0.001 vs. PAE male, determined by *t*-test for independent samples.

Interestingly, an increased *ACE2/ACE* mRNA ratio in the hippocampus was observed only in PAE females (1.13 ± 0.04) compared to the control group (0.92 ± 0.02, *p* ≤ 0.05). (Figure 1A). PAE males showed a reduction in *ACE2/ACE* mRNA ratio (0.51 ± 0.03) in the HC compared to control (0.73 ± 0.04, *p* ≤ 0.001) and PAE females (Figure 1A). The *ACE2/ACE* mRNA ratio increased in the PAE myocardium (0.42 ± 0.03 to males and 0.98 ± 0.02 to females) compared to the control groups (0.34 ± 0.03, *p* ≤ 0.05 and 0.62 ± 0.03, *p* ≤ 0.001, respectively, Figure 1B).

The mRNA expression of B1 receptor expression was strongly induced by alcohol exposure in hippocampal samples from PAE male mice (3.67 ± 0.43) compared to the PAE female group (1.31 ± 0.11, *p* ≤ 0.001, Table 3). Interestingly, in PN45, myocardial B1 mRNA expression remained low in all experimental groups (Table 4). There was a significant increase in B2 receptor mRNA expression in PAE females compared to PAE males in both tissues (MC: 5.39 ± 0.64 vs. 2.32 ± 0.34, *p* ≤ 0.001 and HC: 4.29 ± 0.35 vs. 3.31 ± 0.17; *p* ≤ 0.05). The myocardial and hippocampal tissue KLK mRNA expressions were increased by PAE, regardless of sex, compared to the respective controls (to males: 1.1- and 1.6-fold, respectively; to females: 1.2- and 1.4-fold, respectively; *p* ≤ 0.05, Table 3 and Table 4).

**Figure 2:**
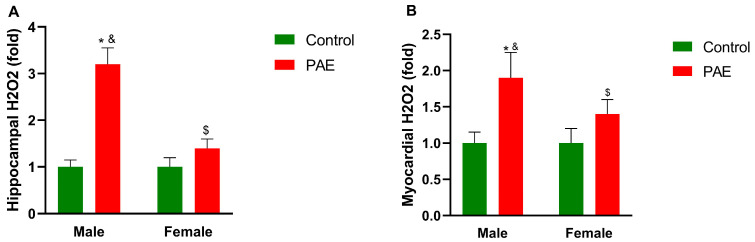
Effect of PAE on the H_2_O_2_ generation in the hippocampus (**A**) and the myocardium (**B**) of animals with PAE of both sexes. Data are shown as mean ± SEM of 4 animals per group. * *p* ≤ 0.001 vs. Control male; ^$^  *p* ≤ 0.001 vs. Control female; ^&^  *p* ≤ 0.001 vs. PAE female, determined by *t*-test for independent samples.

PAE increased the amounts of hydrogen peroxide in both tissues. Heightened content of H_2_O_2_ in PAE males compared to the control (3.2-fold, *p* ≤ 0.001) was observed in the hippocampus (Figure 2A). In the myocardium, H_2_O_2_ augmentation in the PAE male group was less substantial compared to the control (1.9-fold, *p* ≤ 0.001, Figure 2B). PAE females showed an increase of 1.4-fold in H_2_O_2_ content compared to control females in both tissues (*p* ≤ 0.05). Therefore, males presented a significant amount of H_2_O_2_ (2-fold, *p* ≤ 0.001) among PAE animals compared to females in MC and HC (Figure 2A,B).

## 4. Discussion

Sex-specific differences in mRNA expression of components of the RAS and KKS in the hippocampus and myocardium of PAE animals were observed in this study. To the best of our knowledge, this was the first study showing PAE’s modulation of these peptidergic systems in brain and heart tissues. Our data suggest that females submitted to PAE may be less susceptible to alcohol’s deleterious effects. In both tissues, more pronounced expressions of injury-related genes, such as *AT1* and *ACE* mRNA, were found in PAE males. Oppositely, augmented protective *AT2* and *B2* mRNA expressions were observed in PAE females. Interestingly, both tissues also presented distinct differences in mRNA expressions of RAS components of control males and females. Furthermore, oxidative stress measurements revealed, in both tissues, that males submitted to PAE presented higher H_2_O_2_ generation than females.

Although the information on the role of these systems and PAE is scarce, several studies have reported the influence of the RAS on alcohol intake. In 1993, Fitts observed that low-dose peripheral administration of the ACE inhibitor captopril increased the intake of ethanol solution [37]. Previously, [33] collected evidence that alcohol intake is inversely related to RAS activity. Recently, [38] associated PAE and RAS expression using a different protocol. The authors observed an increase in the serum level of Ang II and the gene expression of the renal enzyme *ACE* in PAE animals. In the same study, *AT2* receptor expression was significantly inhibited in the kidneys [38].

Significant upregulation of the *AT1* mRNA in PAE male mice compared to females was found in our study. Analyzing rat hippocampus after the induction of epilepsy by pilocarpine, [39] observed an increase in *AT1* mRNA expression in the chronic phase of the model, where spontaneous and recurrent epileptic seizures are noticed due to the previous formation of hippocampal sclerosis. According to the authors, *AT1* mRNA expression was induced by the injury and consequent neuroinflammation after the pilocarpine insult. Our data suggest that increased hippocampal *AT1* and *ACE* mRNA expressions may reflect increased Ang II availability and activation, worsening the PAE effects, mainly in males.

More recently, [40] reported that a 5% alcoholic diet PAE increased Ang II myocardium concentration and higher apoptotic index in the offspring. The more pronounced gene expression of the *AT1* after PAE is seen in both males’ tissues, which is in consonance with data that noticed *AT1* mRNA augmentation after tissue insults, such as myocardial infarction and cardiac hypertrophy [41,42]. Additionally, [39] reported that *AT2* mRNA expression increased after pilocarpine-induced status epilepticus to protect from damage, contributing to hippocampal plasticity and reorganization of the neuronal network. In our protocol, using a 10% alcohol solution, females submitted to PAE presented decreased *AT1* mRNA expression (compared to PAE males) and a restored *AT2* mRNA expression (compared to the control). These data indicated that alcohol exposure during gestation might be less harmful to females than males.

In male rats with PAE, no differences in ACE mRNA expression were detected in the kidney, but the authors found a diminished ACE2 mRNA expression in males compared to control [38]. Our experiments revealed an increased hippocampal expression of ACE2 mRNA and a higher ratio of ACE2 over ACE mRNA in PAE females than in males. We suggest that this sex difference may be associated with protective properties of ACE2, such as reduced apoptosis [43,44]. Myocardial expression of *ACE* and *ACE2* mRNAs showed different expression profiles between sexes after PAE. In PN45, while an increase in ventricular *ACE* mRNA was observed only in PAE males, *ACE2* transcripts showed higher expression levels in PAE females. A study from [44] reported that, in the myocardium, inflammation caused an increase in *ACE* mRNA expression and decreased *ACE2* mRNA levels in male rats. Thus, in our study, we speculated that PAE could induce a long-lasting inflammatory state that increases injury-related mRNA peptides, especially in males. Therefore, females could be more prone to protection against PAE than males. Moreover, in vitro, alcohol increases *renin* mRNA expression in a concentration-dependent manner in cardiac fibroblasts [45]. The authors observed an increased renin expression in rat hearts induced by alcohol consumption. In our protocol, PAE induced an increased myocardial *renin* mRNA expression in males compared with females.

The observed sex-related differences in the pathophysiology of cardiovascular disease may be driven by androgens, such as testosterone, through the Ang II-ACE-AT1 axis stimulation to induce vasoconstriction, vascular dysfunction, and cardiac dysfunction hypertrophy and fibrosis [46,47]. Some authors observed that estrogen treatment reduced *ACE* mRNA expression and activity in oophorectomized rats’ kidneys, aorta, and lungs [48]. Our data suggest that PAE induced heightened expression of the *ACE-AT1* axis, possibly increasing Ang II levels in the hippocampus and myocardium. The increased *ECA2* mRNA expression in both tissues suggests a protective response through alcohol toxicity. Still, damage-related mRNA expressions induced by PAE were less pronounced in females than in males, which can be partially mediated by sex hormones [49]. Previous studies suggested that Ang II levels are decreased by the suppression of renin and ACE activity by estrogen, thus reducing the activation of the Ang II–ACE–AT1 pathway [50,51].

Interestingly, under physiological conditions, we observed differences in gene expression of RAS components between males and females from the control groups. Other authors had already observed this sexual dimorphism [52,53], showing that male rats have higher AT1 receptor expression in the myocardium, while females tend to have higher AT2 receptor expression. Indeed, in the hippocampus and the LV of control groups, we observed that mRNA expression for the angiotensin *AT1* receptor was lower in females than in males. Sampson et al. [54] demonstrated that males had a trend of an increase in myocardial *AT1* mRNA expression during early adulthood. Wang et al. [55] showed an increased renal expression of the *AT1* receptor mRNA in male mice compared to female control. These authors also found, in the kidneys, higher expression of *AT2* in female control animals than in males. Furthermore, [56] showed that rat females presented increased *AT2* receptor mRNA expression in neurons and that this receptor is required for neurogenesis. Altogether, our data suggested that angiotensin receptors presented distinguished sex-related expression in the hippocampus and LV.

In the hippocampus, we did not detect differences in *ACE* mRNA expression in control males and females. However, some authors have reported sexual dimorphism in *ACE* gene expression in humans. For example, in children, ACE activity is higher in boys and lower in girls after puberty [57]. In healthy young adults, ACE activity is higher in men [58]. Gembardt et al. [59] reported that the distribution of ACE2 is smaller than that of ACE, expressed in the heart, kidneys, lung, brain, intestine, testes, spleen, and adipose tissue in rodents. As the *ECA2* (and *AT2*) genes are located on chromosome X, it is reasonable that there is a sex difference in their expression [60]. The differential expression of *ACE2* between sexes was observed by [61]. Low-fat fed C57BL/6 male mice had higher *ACE2* mRNA expression in kidney and adipose tissue compared to female mice [61]. Our data, analyzing the same strain of mice, showed increased expression of *ACE2* mRNA in the hippocampus of females in the control group compared to males. Moreover, in the LV, females presented increased *ACE2* mRNA expression than males, supporting the work of [61].

Human *renin* mRNA expression has been detected in many organs, such as kidneys, muscles, heart, and brain [62]. Adult mice presented *REN* mRNA expression in the cortex, thalamus, and hippocampus [63]. Renin levels were lower in women than in men [64]. Many studies suggest that estrogen reduces renin secretion from renal juxtaglomerular cells, decreasing plasma renin concentration in humans, while testosterone increases serum renin concentration [65]. Hypertensive animals exhibited sex differences in renin expression [66]. The authors observed that male rats had higher serum renin concentrations than females. Another study analyzing the submandibular gland found that renin levels increased after puberty to become higher in males than in females, and castration reduced renin levels [67]. Other authors have suggested that androgens decrease plasma renin concentrations through estrogenic effects and increase plasma [50]. Accordingly, our data suggest lower expressions of *REN* mRNA in the female mice hippocampus, regardless of PAE.

Unlike the RAS, we did not find differences in hippocampal kinin receptor mRNA expression between male and female control animals. However, the increased myocardial expression of the *B2* receptor seen in the female control mice corroborates a study by [68], showing an increase in *B2* receptor mRNA expression in the left ventricle of female Wistar rats compared to males. Moreover, our data revealed that PAE increases myocardial *B2* receptor mRNA expression in PAE female mice compared to males. Tschöpe et al. [69] found that the cardiac *B2* mRNA levels increased time-dependent after myocardial infarction. The authors proposed that *B2* augmentation is related to cardioprotection exerted by kinin generation and release. We observed that PAE animals presented increased *B1* and *B2* mRNA expressions in the hippocampus. Studying the hippocampus of epileptic patients, [70] observed increased human *B2 receptor* expression in the tissue. Using a pilocarpine-induced epilepsy model, [71] observed an increase in the mRNA expression of kinin receptors in epileptic animals, concluding that the expression of *B1* was related to the insult and the expression of *B2* was increased to counterbalance the deleterious effects to neurons and protect against tissue damage.

Kinin B1 receptor expression is induced under pathological conditions [72]. Marceau et al. [73] reported that the *B1* receptor expression is increased after cell damage and inflammation. After myocardial infarction, high levels of *B1* receptor expression were observed in the acute phase in male rats [74]. Interestingly, myocardial *B1* receptor mRNA expression was unaltered in PAE animals. This observation may be due to the induction of *B1* receptor expression, and the time of euthanasia applied in our protocol. Tschope and colleagues [74] reported that *B1* receptor mRNA decreases gradually after myocardial infarction. In addition, [75] observed temporal differences in kinin receptors mRNA expression in post-infarction phases in Wistar rats of both sexes. A recent study [76] reported the causal role of the B1 receptor in neuroinflammation and oxidative stress in primary hypothalamic neurons. Although we verified an increase in the expression of B1 receptor mRNA in the hippocampus of PAE animals of both sexes, in males, this increase was substantially higher than in females. Some authors [77] reported the mRNA expression of *tissue kallikrein* in developing rat brains, with a peak expression on the first postnatal day. *KLK* mRNA expression was maintained high until postnatal day 10 and gradually decreased. Kallikrein infusion in the myocardium attenuated inflammation and reduced oxidative stress in rats after MI [78]. The authors observed that kallikrein and B2 receptors activation induced the suppression of oxidative stress after MI. Previous work from Iwadate et al. [79] found that KLK protein content in rat brains continuously decreased after birth until PN49. According to our findings, PAE induced an increase in hippocampal *KLK* mRNA expression in animals of both sexes exposed to alcohol during pregnancy at PN45. A study with the human hippocampus observed that neuroinflammation increased *KLK* mRNA and protein expressions in refractory temporal lobe epilepsy [80]. Other authors have observed differences in kallikrein expression between sexes in other organs. In the kidneys, *KLK* mRNA expression was higher in female rats than in males. However, no difference was found in the heart among the control groups [68]. This observation corroborates the data obtained in our study, which unaltered myocardial *KLK* mRNA expression differences between sexes from control groups. Nevertheless, PAE increased *KLK* mRNA expression only in the myocardium of PAE female mice. Silva-Jr et al. [81] reported that rats bearing human tissue kallikrein transgene showed marked cardioprotection to cardiac hypertrophy and fibrosis. In agreement with previous works [81,82], our data suggest that KKS activation by incremented *B2* and *KLK* expression may result in cardioprotection toward damage induced by PAE.

Smith et al. [83], quantifying malondialdehyde (MDA) levels in the rat cerebellum, hippocampus, and cortex, observed high concentrations in the cerebellum of animals that received alcohol. In the hippocampus and cortex, however, MDA levels were unaffected by alcohol treatment [84]. Increased oxidative stress induced by PAE in developing organs has been reported by several studies [22,85,86]. Increased NADPH oxidase mRNA was detected in the cerebellum of PAE rats [84]. Moreover, lower expressions of superoxide dismutase, glutathione peroxidase, and catalase were detected in fetal brains exposed to alcohol [86]. The increased amount of hydrogen peroxide in the tissues of PAE animals may reflect the high cellular alcohol-induced ROS in these animals. O_2_ and H_2_O_2_ concentrations are regulated by the cellular antioxidant system and physiologically can activate signal transduction pathways [85]. However, if a large ROS production exceeds the antioxidant protection ability, the generation of oxidative stress induces several intracellular damages [87]. In our protocol, the 10% alcoholic solution was administered through the whole gestation and weaning, allowing the teratogenic effect of alcohol to be present during embryonic development. As a result, PAE groups presented a heightened myocardial H_2_O_2_ concentration, although higher levels were observed in males than in females. A review study [88] reported several antioxidant interventions against FASD targeting oxidative stress in animal models and speculated that more clinical trials are needed to evaluate their efficacy in humans with PAE.

The fetus’s endogenous antioxidant system is less active than in adult mice and consequently more vulnerable to alcohol toxicity [89]. Given the increased susceptibility of the brain to alcohol effects than other organs [90], a marked increase in hippocampal H_2_O_2_ concentration was found in males compared to females of PAE groups. A previous study by [91] showed that females generate lower levels of H_2_O_2_ in cardiac and brain tissues; however, we did not detect differences in H_2_O_2_ content in control animals in both tissues. These findings about control animals also contradict [92], which reported that male rats produce more ROS than age-matched females. These discrepancies may have two reasons; the limited sample size utilized in our study, or the different methodology applied in the cited studies.

Three significant limitations should be considered. First, although we performed mRNA quantifications, this study did not address protein translation. Measurements of G protein-linked transmembrane receptors are a challenge in the literature, primarily due to nonspecific commercial antibodies available [93]. Secondly, this was a cross-sectional study. A temporal gene expression of these peptidergic systems may facilitate understanding the findings achieved. Our lab is running experiments to address this topic, and we hope to show these data soon. A previous study [22] analyzed the activation of several signal transduction pathways in the myocardium at a different time point than in this study. Another limitation was the measurement of oxidative stress by determining the H_2_O_2_ content. Therefore, other techniques will be applied to increase the knowledge of the involvement of oxidative stress in PAE.

In summary, PAE altered the mRNA expression of several genes of the RAS and KKS in the hippocampus and myocardium. As a proposal pathway, to counteract the effects of *AT1/ACE/B1* expression, tissue protection against PAE may occur partly by expressing protective biomarkers such as *AT2*, *B2*, *KLK*, and *ACE2* mRNA, especially in PAE females. In addition, diminished cellular ROS levels found in PAE females suggested a reduced stressor effect of alcohol in the hippocampus and LV of PAE females.

## 5. Conclusions

PAE modulated hippocampal and myocardial expressions of genes in the renin–angiotensin and kallikrein–kinin peptidergic systems. Sex-specific differences in mRNA expression of these peptides in both tissues were found with or without gestational alcohol exposure. A protective modulation of these systems in PAE females was mightily indicated, along with lower levels of ROS found in the hippocampus and the myocardium. ACE inhibitors, whose efficacy in tissue protection has been evidenced by several authors [94,95,96], could be considered to decrease the myocardial and hippocampal damage effects of PAE.

## Figures and Tables

**Table 1:** Effects of PAE on the transcription modulation of myocardial renin–angiotensin system genes in males and females.

	Control		P.A.E.	
	Male	Female	Male	Female
*AT1*	4.72 ± 0.51	3.48 ± 0.39 ^a^	8.78 ± 1.23 ^aa^	4.28 ± 0.32 ^b,c^
*AT2*	0.92 ± 0.21	1.57 ± 0.31 ^a^	1.21 ± 0.16	2.44 ± 0.41 ^b,c^
*REN*	3.43 ± 0.28	2.96 ± 0.21	5.82 ± 0.86 ^a^	3.53 ± 0.30 ^b,c^
*ACE*	3.79 ± 0.81	3.99 ± 0.54	7.63 ± 0.92 ^aa^	4.27 ± 0.41 ^c^
*ACE2*	1.30 ± 0.33	2.49 ± 0.23 ^a^	3.28 ± 0.34 ^a^	4.19 ± 0.46 ^b,c^

Data are shown as mean ± SEM of 5 animals per group. ^aa^  *p* ≤ 0.001 vs. Control male; ^a^  *p* ≤ 0.05 vs. Control male; ^b^  *p* ≤ 0.05 vs. Control female; ^c^  *p* ≤ 0.05 vs. PAE male.

**Table 2:** Effects of PAE on the transcription modulation of renin–angiotensin system genes in the hippocampus of males and females.

	Control		P.A.E.	
	Male	Female	Male	Female
*AT1*	3.43 ± 0.41	1.92 ± 0.53	9.91 ± 1.46 ^aa^	3.43 ± 0.69 ^b,c^
*AT2*	1.01 ± 0.18	1.34 ± 0.31 ^a^	1.30 ± 0.15 ^a^	2.77 ± 0.46 ^b,c^
*REN*	2.71 ± 0.72	2.19 ± 0.83	2.94 ± 0.68	2.63 ± 0.33
*ACE*	4.12 ± 0.84	3.64 ± 0.27 ^a^	5.68 ± 0.50 ^a^	4.28 ± 0.23 ^b,c^
*ACE2*	3.01 ± 0.17	3.38 ± 0.21 ^a^	2.93 ± 0.57 ^a^	4.87 ± 0.77 ^b,c^

Data are shown as mean ± SEM of 5 animals per group. ^aa^  *p* ≤ 0.001 vs. Control male; ^a^  *p* ≤ 0.05 vs. Control male; ^b^  *p* ≤ 0.05 vs. Control female; ^c^  *p* ≤ 0.05 vs. PAE male.

**Table 3:** Hippocampal mRNA expression of components of the kallikrein–kinin system in males and females submitted to PAE.

	Control		P.A.E.	
	Male	Female	Male	Female
*B1*	0.32 ± 0.27	0.43 ± 0.40	3.67 ± 0.43 ^a^	1.31 ± 0.11 ^b,cc^
*B2*	2.73 ± 0.23	3.28 ± 0.32	3.31 ± 0.17	4.29 ± 0.35 ^b,c^
*KLK*	2.47 ± 0.37	3.65 ± 0.90	3.89 ± 0.81 ^a^	4.98 ± 0.33 ^b^

Data are shown as mean ± SEM of 5 animals per group. ^a^  *p* ≤ 0.05 vs. Control male; ^b^  *p* ≤ 0.05 vs. Control female; ^cc^  *p* ≤ 0.001 vs. PAE male; ^c^  *p* ≤ 0.05 vs. PAE male.

**Table 4:** Myocardial modulation of mRNA expression of the kallikrein–kinin system genes in PAE males and females.

	Control		P.A.E.	
	Male	Female	Male	Female
*B1*	0.86 ± 0.21	0.99 ± 0.40	1.12 ± 0.48	1.07 ± 0.37
*B2*	2.18 ± 0.69	2.29 ± 0.41	2.32 ± 0.34	5.39 ± 0.64 ^b,cc^
*KLK*	3.48 ± 0.28	4.09 ± 0.37	3.90 ± 0.92 ^a^	4.95 ± 0.36 ^b^

Data are shown as mean ± SEM of 5 animals per group. ^a^  *p* ≤ 0.05 vs. Control male; ^b^  *p* ≤ 0.05 vs. Control female; ^cc^  *p* ≤ 0.001 vs. PAE male.

## Data Availability

Data are contained within the article and Supplementary Materials. More data are available upon request to the corresponding author, J.A.S.J.

## Supplementary Materials

The following supporting information can be downloaded at: 10.5281/zenodo.7496613 (accessed on 2 January 2023). Table S1. Target genes and sequences of the primers used for qPCR.
